# Comparison of spatiotemporal gait parameters and their variability in typically developing children aged 2, 3, and 6 years

**DOI:** 10.1371/journal.pone.0285558

**Published:** 2023-05-11

**Authors:** Markéta Rygelová, Jaroslav Uchytil, Isaac Estevan Torres, Miroslav Janura

**Affiliations:** 1 Department of Human Movement Studies, University of Ostrava, Ostrava, Czech Republic; 2 Department of Teaching Corporal Expression, University of Valencia, Valencia, Spain; 3 Faculty of Physical Culture, Palacky University Olomouc, Olomouc, Czech Republic; Universidad Nacional Autonoma de Mexico, MEXICO

## Abstract

Independent walking is an important milestone in a child’s development. The maturation of central nervous system, changes in body proportions, spatiotemporal parameters of gait and their variability change are dependent on age. The first aim of this study was to compare non-normalized and normalized spatiotemporal parameters and their variability in children. The second aim was to determine which spatiotemporal parameters are most affected by aging. Data from 64 typically developing children (age: 2.0–6.9 years), who walked at a self-selected speed along a 10m walkway, were collected with a motion capture system. Spatiotemporal parameters were normalized based on leg length. The main effect of the non-normalized walking speed revealed a moderate effect size (ES = 0.72) comparing 2- and 3-years-old, a large effect size comparing 2- and 6-years-old (ES = 1.77), and a large ES comparing 3- and 6-years-old (ES = 1.22). The normalized stride width parameter showed a statistically significant difference with large effect size between 2 vs 3 (ES = 1.00), 2 vs 6 (ES = 3.17), and 3 vs 6 (ES = 1.96). A statistically significant decrease in intra-individual gait variability with increasing age was observed in all parameters except for stride width. The variability of stride width may serve as a parameter in 2-year-olds to assess deviations from typically developing children. The assessment of effect size could be a useful indicator for clinical practice.

## Introduction

An important milestone in a child’s development is the initiation of independent walking, that is, the child’s transition from sitting to unsupported walking [1]. During the early years, the central nervous system (CNS) matures in childhood and dynamic balance is affected by the learning process which contributes to the development of gait [2, 3]. In children who are preschoolers, immature postural and locomotor control results in unstable movements [4, 5]. Gait at this age is characterized by a wide base of support, small steps and a predominant double support phase. The high cadence and stride length variability is due to the effort to maintain balance [6, 7]. In children aged 3-4 years, the dynamic joint angle measurements indicate stabilization of gait is similar to that of adults [2]. However, spatiotemporal parameters (STP) begin to stabilize at approximately 7 years of age [4, 7–9]. The changes in STP related to the maturation of gait are influenced by the maturation of the musculoskeletal system and experience with unsupported walking [5, 10].

Body height increases with age and affects spatial parameters (step length and stride width), additionally, walking speed also increases with age and affects temporal parameters (stance time, cycle time, step time, swing time, double support time) [2, 11]. Different approaches can be used to compare gait between different age groups of children with different walking speeds. For example, a fixed treadmill gait speed is often used, but in this case, it is not a natural gait [12]. Another option is to walk at self-selected speed and then normalize the results using the Froude number [13–15]. The normalization of STP using leg length or body height minimizes inter-individual differences associated with the unequal weight and stature of the different subjects [16]. Studies on pediatric gait often compare the non-normalized or normalized STP between age groups. Guffey et al. [13] compared non-normalized and normalized STP by leg length in children aged 2-4 years and observed a decrease in non-normalized cadence for age groups and an increase in non-normalized stride length with increasing age. However, the authors [13] did not find any significant differences between age groups in the normalized STP. Similarly, in South African children, no differences were found in normalized STP between the age groups 6-8 and 9-10 years [17]. In contrast, Mexican children aged 6-13 years exhibited a decrease in the number of steps, normalized walking speed, walking speed, cadence, normalized cadence, normalized step and stride length and increased step and stride length with increasing age [18]. Furthermore, in French children aged 6-12 years, speed, step length and stride length increased with increasing age [19]. Cadence decreased and step time, cycle time and stance time increased significantly between 6 and 7 years of age compared to 9 years and older. However, the observed values of STP are in conflict with studies from other countries [19]. In summary, non-normalized stride length and gait speed increase with increasing age, but there is a decrease with age in the normalized gait speed, non-normalized and normalized cadence and stride length variability [11].

Various authors compared children with different body heights using STP normalization [11, 16, 20]. The most reported parameter considering CNS maturation was children´s age such that, with increasing age, gait variability decreases [21, 22]. Thus, if we aim to describe the differences in CNS maturation independent of body height, we should focus on those parameters where variability decreases with age and their normalized values are different. However, there has been little discussion about STP parameters that are significantly affected by maturation age differences [4, 21].

Therefore, the purpose of this study was to compare non-normalized and normalized STP and their variability in children aged 2, 3 and 6 years of age. We hypothesized that there will be changes in normalized STP between 2- and 3-year-old children based on prevailing age changes; between 2- and 6-year-old, and between 3- and 6-year-old children and that there will be more changes in non-normalized STP due to maturation. Additionally, we hypothesized that variability in STP in all age categories will change because STP are more dependent on CNS development.

## Methods

### Ethics statement

This study was approved by the Ethical Board of the University of Ostrava, Faculty of Education (protocol ID: OU-54483/45-2019) and was conducted by the principles of the Declaration of Helsinki. The parents were asked to provide signed consent to confirm the participation of their children in the study before the start of the experiment.

### Participants

Sixty-four typically developing, preschool children aged 2, 3 and 6 years participated in this cross-sectional study. Anthropometric data are presented in Table 1 and participants were divided into three age groups. The exclusion criteria included any injury, developmental disorder or other abnormalities associated with gait deformities in children included in the study. At the time of data collection, parents did not report any health problems in their children. Data collection took place between September 2019 and December 2021 in the biomechanics laboratory of the Human Movement Diagnostic Centre (University of Ostrava).

**Table 1:** Participant characteristics (Mean ± SD) for each age group.

Group	N	Age (y)	Sex (M: F)	Mass (kg)	Height (cm)	First steps (months)
2 (2–2.9 y)	20	2.4 ± 0.2	10:10	13.3 ± 1.2	93.2 ± 4.5	12.5 ± 1.9
3 (3–3.9 y)	22	3.5 ± 0.3	7:15	15.3 ± 2.9	98.9 ± 5.0	12.5 ± 2.0
6 (6–6.9 y)	22	6.4 ± 0.3	11:11	22.4 ± 3.6	120.2 ± 5.3	12.6 ± 2.1

Note. N = number of children, F = female, M = male, y = years, First steps—age of first unsupported steps.

### Experimental set-up

Three-dimensional kinematics were sampled at 240 Hz using 10 cameras (Qualisys, 9 x Oqus 700, 1 x Oqus 510, Sweden) motion capture system (Qualisys AB, Gothenburg, Sweden). Twenty-two 6.4 mm retro-reflective markers and four specific landmark markers were placed on the pelvis and lower limbs. Markers were placed bilaterally on the posterior superior iliac spines, anterior superior iliac spines, greater trochanter, lateral and medial femoral epicondyles, lateral and medial malleoli, calcaneus, 1st metatarsal, 5th metatarsal, position point on the lateral side of the leg and four specific landmarks on the shank and thigh according to the recommendation of Visual 3D (Visual 3D, C-motion, Rockville, MD, USA).

### Protocol

Participants visited the laboratory on two occasions. In the first visit, each child was introduced to the laboratory and the measurement protocol. Parents were asked about their child’s health, previous musculoskeletal injuries and age at which they started walking. In the second visit, before the measurement began, anthropometric data (leg length, body height and weight) were obtained from the child. Each child walked on a 10-meter-long walkway marked with coloured cones. The child was told “You can go” and they walked between their parents, each standing at one end of the walkway. Three walking practice attempts were performed, followed by 12 complete barefoot trials at the child’s self-selected speed. If any of the children stopped, started running, or exhibited any other unusual behavior that did not correspond to walking, the trial was excluded. All data were collected by the same examiner.

### Data analysis

Data were analyzed with Qualisys Track Manager (Qualisys AB, Gothenburg, Sweden) and Visual 3D software (C-motion, Rockville, MD, USA). For each child, a personalized model based on the child’s anthropometric measurements was created. Several non-normalized and normalized STP by leg length trials were selected for each complete stride which are commonly used in clinical practice (cycle time, double limb support time, stride width, stance time, step length, swing time and walking speed) [21]. Leg length was determined as the distance between markers of the greater trochanter and the lateral malleolus. Spatiotemporal parameters were normalized by leg length [16]. Gait variability was determined using the coefficient of variation (CV,[%]) for each participant for both lower limbs together and for left and right lower limbs separately [22]. Walking speed was normalized with the Froude number for each age group to assess differences between the groups according to participants’ body height and self-selected speed [16]. The Froude number is calculated as v^2^/*g* * *l*, where v = velocity, g = the acceleration due to gravity and l = leg length or the distance from the greater trochanter to the lateral malleolus. The Froude number is used to compensate for differences in velocity, body size and gravity [23].

### Statistical analysis

Data management and analysis was performed using IBM SPSS 24 (Armonk, NY, USA: IBM Corp). Descriptive statistics (mean and SD) were computed for all STP. Normalized STP were calculated according to [16]. Normal distribution was assessed by the Shapiro-Wilk test. Data normality was confirmed for leg length, Froude number values and all STP. Data normality was not confirmed for Intra-individual variability (CV). For variables where the normality of the data distribution was confirmed, a one-way Analysis of Variance (ANOVA) with a criterion significance level at p = 0.05 was used for a between age group comparison. If a significant main effect was observed, a post hoc test was performed. Bonferroni post hoc tests were used for pairwise comparisons with significance level for multiple comparisons set at p = 0.02. If the data were not normally distributed, a non-parametric Kruskal-Wallis test was used to compare the age groups. To assess intra-individual variability, a CV was calculated for each individual and each STP.

Cohen’s d was calculated for the effect size (ES) and used to quantify the difference between age groups for STP and intra-individual variability [24]. Cohen’s d thresholds (<0.2 is trivial, 0.2-0.6 is small, 0.6-1.2 is moderate, 1.2-2.0 is large, 2.0-4.0 is very large, >4.0 is nearly perfect) were employed [25].

## Results

### Non-normalized and normalized spatiotemporal parameters

The mean and SD values of the non-normalized and normalized STP and the differences between age groups are show in Table 2. A statistically significant difference in non-normalized speed and non-normalized step length between the age groups 2- and 6-years-old (p <0.001), 3- and 6-years-old (p <0.001) was found. However, the difference in non-normalized speed between 2- and 3-years-old children was only of moderate effect size (ES = 0.72), for non-normalized step length there were large age effect between all age categories (*ES*_2−3_ = 1.33, *ES*_2−6_ = 4.21, *ES*_3−6_ = 3.11). For the rest of the non-normalized parameters, except for double support time, six-year-old children showed higher values than the other two groups in stance time, cycle time, step time, swing time (p <0.001). Double support time revealed that there were no statistically significant differences between 2- and 6-years-old children (p = 0.04); but statistically significant differences with moderate effect size were found between 3- and 6-years-old (p <0.02; ES = 1.18). With the exception of normalized stride width, there were no statistically significant differences and only trivial effect sizes across the normalized STP parameters. Large effect size (ES = 1.28) was found for the normalized double support time between 2- and 6-years-old. There was a statistically significant difference in normalized stride width between 2- and 6-years-old and 3- and 6-years-old (p <0.001) with moderate effect size (*ES*_2−6_ = 3.17; *ES*_3−6_ = 1.96), likewise between age categories 2- and 3-years-old (p = 0.003; ES = 1.00).

**Table 2:** Non-normalized and normalized spatiotemporal parameters between age groups.

Variables	Group 2	Group 3	Group 6	ES^*Sig*.^ 2–3	ES^*Sig*.^ 2–6	ES^*Sig*.^ 3–6
Walking Speed (m/s)	0.90 ± 0.14	0.99 ± 0.11	1.13 ± 0.12	0.72*	1.77**	1.22**
Normalized	0.23 ± 0.07	0.24 ± 0.05	0.25 ± 0.05	0.17^*NS*^	0.33^*NS*^	0.20^*NS*^
Stance time (s)	0.49 ± 0.06	0.49 ± 0.04	0.56 ± 0.04	0.00^*NS*^	1.39**	1.75**
Normalized	0.26 ± 0.03	0.25 ± 0.02	0.25 ± 0.02	0.40^*NS*^	0.40^*NS*^	0.00^*NS*^
Cycle time (s)	0.75 ± 0.09	0.76 ± 0.06	0.87 ± 0.07	0.13^*NS*^	1.50**	1.69**
Normalized	0.39 ± 0.05	0.38 ± 0.03	0.38 ± 0.03	0.25^*NS*^	0.25^*NS*^	0.00^*NS*^
Step length (m)	0.34 ± 0.03	0.38 ± 0.03	0.49 ± 0.04	1.33*	4.21**	3.11**
Normalized	0.91 ± 0.08	0.92 ± 0.06	0.93 ± 0.08	0.14^*NS*^	0.25^*NS*^	0.14^*NS*^
Step time (s)	0.38 ± 0.04	0.38 ± 0.03	0.44 ± 0.03	0.00^*NS*^	1.71**	2.00**
Normalized	0.20 ± 0.02	0.19 ± 0.02	0.19 ± 0.01	0.50^*NS*^	0.64^*NS*^	0.00^*NS*^
Swing time (s)	0.26 ± 0.03	0.27 ± 0.02	0.31 ± 0.02	0.40^*NS*^	1.98**	2.00**
Normalized	0.14 ± 0.01	0.13 ± 0.01	0.13 ± 0.01	1.00^*NS*^	1.00^*NS*^	0.00^*NS*^
Double support time (s)	0.24 ± 0.03	0.23 ± 0.02	0.26 ± 0.03	0.40^*NS*^	0.67^*NS*^	1.18*
Normalized	0.13 ± 0.02	0.12 ± 0.01	0.11 ± 0.01	0.64^*NS*^	1.28^*NS*^	1.00^*NS*^
Stride width (m)	0.09 ± 0.01	0.08 ± 0.01	0.08 ± 0.01	1.00^*NS*^	1.00^*NS*^	0.00^*NS*^
Normalized	0.23 ± 0.03	0.20 ± 0.03	0.15 ± 0.02	1.00*	3.17**	1.96**

Note. ES = effect Size, Sig. = difference between groups at *p <0.02, **p <0.001, NS = No Significance.

### Intra-individual variability of spatiotemporal parameters

**Fig 1:**
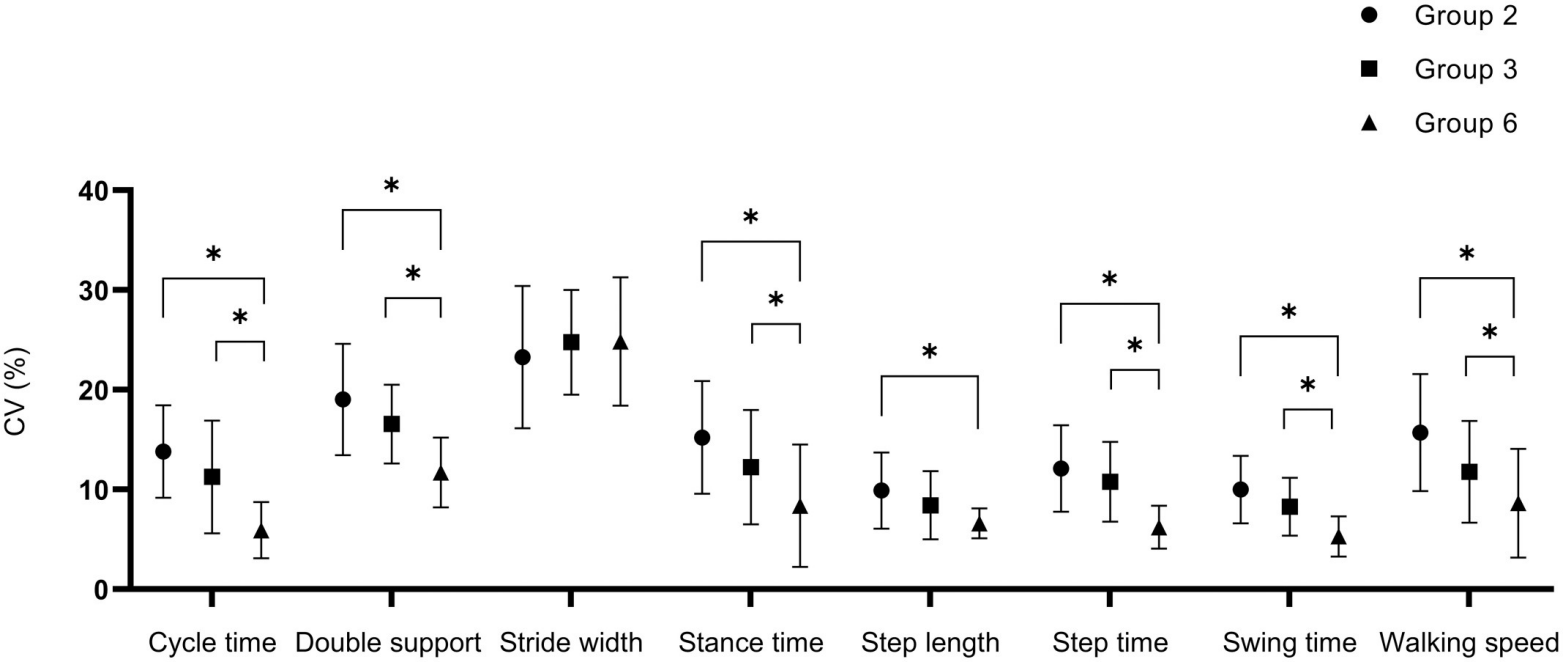
Intra-individual variability of spatiotemporal parameters. All data reported for both limbs as Mean ± SD. Note. CV = Coefficient of variation; * p<0.05.

There were statistically significant differences (p <0.001)) among the age groups for all intra-individual variabilities of the STP parameters, except for stride width and step length Fig 1. All parameters, except for stride width, showed a decrease in CV with increasing age.

Similar effects to those shown in Fig 1, both lower limbs together were similar for the left and right lower limbs separately (Table 3). The effect of age group was observed for stance time (left and right (p <0.001), cycle time (left and right p <0.001), step time (left and right p <0.001), swing time (left and right p <0.001), step length (left p = 0.029 and right p <0.001) and walking speed (p <0.001). Mainly, the 6-year-old group exhibited lower CV than the 2- and 3-year-old.

**Table 3:** Intra-individual variability for left and right lower limbs between age groups.

CV (%)	Group 2	Group 3	Group 6	ES^*Sig*.^ 2–3	ES^*Sig*.^ 2–6	ES^*Sig*.^ 3–6
Left						
Stance time	15.05 ± 5.51	12.09 ± 5.54	7.46 ± 5.07	0.54^*NS*^	1.44**	0.87**
Cycle time	13.82 ± 6.30	10.76 ± 6.43	6.20 ± 3.77	0.48^*NS*^	1.49**	0.87**
Step length	10.21 ± 4.43	7.99 ± 2.94	6.79 ± 1.65	0.60^*NS*^	1.04*	0.50^*NS*^
Step time	11.82 ± 4.41	10.23 ± 3.42	6.06 ± 2.20	0.41^*NS*^	1.68**	1.45**
Swing time	10.06 ± 3.75	7.70 ± 1.95	5.21 ± 1.82	0.80^*NS*^	1.67**	1.32**
Right						
Stance time	15.42 ± 5.91	12.39 ± 6.04	7.68 ± 4.96	0.51^*NS*^	1.42**	0.85**
Cycle time	13.18 ± 5.12	11.16 ± 5.91	5.45 ± 2.47	0.36^*NS*^	1.95**	1.26**
Step length	9.78 ± 3.23	8.85 ± 3.85	6.39 ± 1.36	0.26^*NS*^	1.39**	0.85^*NS*^
Step time	12.40 ± 4.38	11.33 ± 4.54	6.43 ± 2.24	0.24^*NS*^	1.74**	1.37**
Swing time	9.97 ± 3.06	8.85 ± 3.56	5.58 ± 2.08	0.34^*NS*^	1.69**	1.12**

Note. CV = Coefficient of variation, ES = Effect Size, Sig. = significant difference these two groups at *p <0.02, **p <0.001, NS = No Significance.

## Discussion

The aim of this study was to compare non-normalized and normalized STP and their variability, and to compare these parameters in normally developing children aged 2, 3 and 6 years to determine which parameters are most affected by age. The results of this study add to previous literature regarding the development of gait in children [11, 13, 17–19], particularly the data on children 2 and 3-years-old that are uncommon. Our study also presented results on the CV in STP and the assessment of differences between age groups.

In our study, data were obtained at a self-selected walking speed. Due to the different body height of children and different walking speed which affects STP [26], the data were normalized [16]. Moreover, in our study, the age of first unsupported steps was similar between age groups (see Table 1) and thus, it can be said that motor development was similar across all age groups. Therefore, we assume that the results presented in this study represent walking of typically developing children.

The results showed that significant changes occurred in the non-normalized and normalized walking speed and step length parameters that increased with aging. On the contrary, stride width decreased with increasing age. For walking speed and step length values, our results were in agreement with the studies by others [7, 13, 27]. However, our results do not agree with other researchers [18, 28]. All of the above studies used a different measurement method, GaitTire, which could explain the different findings [7, 13, 18, 27, 28]. However, we suggest that the differences are more likely attributable to the different distribution of the age groups studied. In some studies, [7, 13, 18, 27, 28] we found only a statistical significance was used to compare differences. In our study, we supplemented the statistically significant differences with the effect size (ES). At least a moderate effect sizes were revealed in the parameters where a statistically significant difference was observed (ES = 0.67—4.21). The assessment of effect size can be a useful indicator for clinical practice, especially for the simplicity of the calculation and rapid interpretation of results. Effect size values greater than 0.6 can be considered significant when assessing deviations from the normal.

Common indicators of walking stability are stride width, stride length and their variability. However, it is not entirely clear to what extent these parameters are affected by walking speed, CNS maturation or height. With increasing age, the non-normalized speed increased in children, while the normalized speed did not differ in the studied age groups. Thus, it is apparent that speed can be affected by height. If we want to compare speed in individuals of the same age and different body height, we need to normalize the speed using the Froude number. Speed variability decreased with age and may be an indicator of gait development in children [22]. There were significant differences in speed variability between children aged 2 and 6 years. The decreasing trend was also evident among children aged 3 and 6 years; however, these changes were not statistically significant. Thus, a faster development of gait stability can be expected in children with less experience of independent walking than in older children. The same trend of decreasing variability was also present for other STP, except for stride width, where this decrease did not occur. There was a significant difference in step length only between children aged 2 and 6 years. Individuals with low stride width variability may lack the skills needed to adjust stride width to be able to maintain and control balance that contributes to the maturation of gait [22, 29]. If we want to assess the differences in stability in children with possible movement difficulties who move at different speeds, stride width and stride width variability can be used. This parameter appeared to be the least dependent on speed and age in children from 2 years of age. However, in previous studies dealing with stride width variability, the effect of speed on stride width variability has not been clearly demonstrated [30, 31]. Further studies are therefore needed for a clearer understanding. Stride width was also associated with gait stability and may be an indicator of gait balance problems and a possible risk of falls in adults [31–33]. This may be the case for children as well, however, this fact has not been sufficiently researched and may be the subject of further studies. On the contrary, the step length and variability step length parameters seemed to be the most speed-dependent. Significant differences in non-normalized step length were found between all age categories as well as in non-normalized walking speed. Similar trends in STP variability have been reported in the other studies and for older children as well [7, 11, 12].

This study has several limitations. Insufficient motivation and maintaining children’s attention could be a limitation that could affect natural walking. This factor was minimized by acclimating children to the laboratory environment, use of a favorite toy and cooperation with parents. Attempts where the child obviously did not walk naturally were ruled out. Lastly, the data obtained in current study are always of a cross-sectional nature. For a more accurate description of the development of gait in typically developing children, it would be beneficial to obtain data from longitudinal studies in future.

## Conclusion

This study has found that, generally, children aged two and three years showed differences in non-normalized STP (walking speed, stance time, cycle time, step length, step time, swing time, double support time) compared to six-year-olds. The difference in these parameters was observed with statistical significance from moderate to very large effect size. Increase in these parameters was in line with increasing age. There is another important finding with the non-normalized stride width parameter where this increasing with age trend was not found. The results showed only a significant difference for normalized stride width which decreased with increasing age. Increasing age also decreased in intra-individual variability for all STP except for stride width, where we did not find a difference in variability. Stride width appears to be a parameter independent of age and body height. It may be an indicator of differences in normal and pathological walking of the subjects compared.
